# A case of two connected stents deployed during iStent inject W surgery

**DOI:** 10.1186/s12886-023-02951-z

**Published:** 2023-05-10

**Authors:** Ayaka Shimada, Sho Ichioka, Akiko Ishida, Sachiko Kaidzu, Masaki Tanito

**Affiliations:** Department of Ophthalmology, Shimane University Faculty of Medicine, 89-1 Enya, Izumo, 693-8501 Japan

**Keywords:** Minimally invasive glaucoma surgery, iStent, Surgical complication

## Abstract

**Background:**

We report a case with two connected stents ejected simultaneously during an iStent *inject* W surgery, a modified second-generation iStent Trabecular Micro-Bypass System.

**Case presentation:**

A 57-year-old woman with primary open-angle glaucoma underwent a combined cataract and iStent *inject* W surgery in her left eye. After the trabecular meshwork/Schlemm’s canal was pierced by the trocar of injector, the delivery button was pressed a first time, but the stent was not ejected. After the button was pressed a second time, connected two stents were ejected. After removing the dislocated stents from the anterior chamber, two stents were implanted into the desired places using another injector. Except for mild hyphema, no postoperative complication occurred. Stereomicroscopic observation showed that the two stents were connected by a broken trocar shaft. An X-ray showed that the trocar shaft was broken at the part referred to as the “sprayed trocar”. Scanning electron microscopy showed that the surface features of the broken trocar and trocar tip represented tensile failure.

**Conclusions:**

Although rare, considering that the damage was seen at the structurally weak part (i.e., sprayed trocar), the same phenomenon can happen. For patient safety, surgeons are recommended to inspect the device when the deployment of either the first or second stent is unsuccessful during the iStent *inject* surgery.

**Supplementary Information:**

The online version contains supplementary material available at 10.1186/s12886-023-02951-z.

## Background

Implantation of a second-generation iStent Trabecular Micro-Bypass System (iStent *inject*, Glaukos Corporation, San Clemente, CA) in combination with cataract surgery is associated both with significant postoperative reduction of intraocular pressure (IOP) compared with cataract surgery alone in eyes with primary open-angle glaucoma (POAG) and few vision-threatening postoperative complications [1]. Currently, the modified second-generation iStent (iStent *inject* W), which has a wider stent flange than the iStent *inject*, is clinically available. We report a case that two connected stents were ejected together during an iStent *inject* W surgery.

## Case presentation

A 57-year-old woman was referred to our department from the gynecology department for preoperative consultation regarding robot-assisted surgery with a head-down position. She had no remarkable ocular medical history. At referral, the best-corrected visual acuity (BCVA) was 0.7 with − 10.5 diopters (D) of myopic correction in the right eye (OD) and 0.9 with − 11.25 D myopic correction in the left eye (OS). The IOPs were 24 mmHg OD and 23 mmHg OS. The anterior chamber (AC) angle was wide open in both eyes (OU); an Emery-Little grade 1 nuclear cataract was observed OU; and the cup-to-disc ratios were 1.0 × 0.8 OD and 0.8 × 0.8 OS. After the successful gynecologic surgery, she visited our department for further ocular evaluation 2 weeks after the initial visit. The visual field mean deviation (MD) was − 8.15 decibels (dB) OD and − 4.73 dB OS, and the foveal sensitivity was 25 dB OD and 34 dB OS using the Humphrey Visual Field Analyzer (Carl Zeiss Meditec, Dublin, CA; central 30 − 2 program). Thinning of the retinal nerve fiber was detected OU by optical coherence tomography (OCT) (RS3000 Advance 2, Nidek, Gamagori, Japan). Based on the findings, she was diagnosed with POAG OU, and ocular hypotensive medication OU was prescribed. Two years later, the BCVA was 0.5 OD and 0.9 OS, and the IOP was 23 mmHg OD and 17 mmHg OS with four classes of medications. Because of insufficient IOP reduction, a triple procedure (microhook trabeculotomy OD and iStent *inject W* OS combined with cataract surgery) was planned [2]. Two days after the uncomplicated surgery OD, a planned surgery was performed OS by one of the authors (MT).

**Fig. 1:**
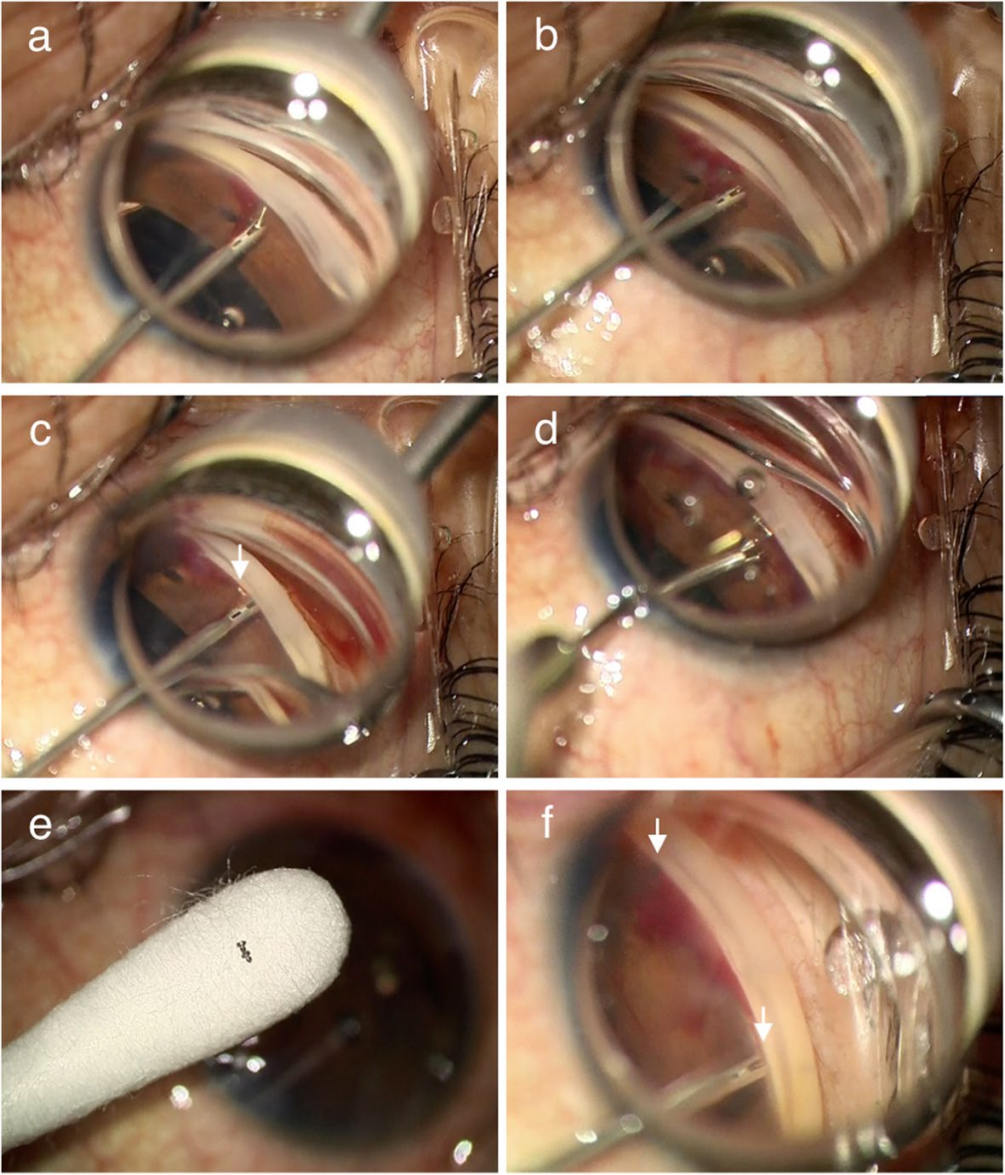
Surgical findings. After the first deployment, the stent is not ejected; the trocar tip appears bent **a**. After release of the injector button, the stent returns into the sleeve **b**. After the second deployment, the stent is dislocated on the iris (**c**, arrow). After removal using forceps (**d**), the stents are observed under surgical microscopy **e**. Using a new injector, two stents are implanted at the nasal angle (**f**, arrows)

After the implantation of a soft acrylic intraocular lens through a 2.2-mm-wide nasal corneal incision, the sleeve of the iStent injector was inserted into the AC through a 1-mm-wide superotemporal corneal side port, that created as a side port during cataract surgery, to place the stents in the nasal-side angle. To visualize the AC angle, a Swan-Jacob gonioprism lens (Ocular Instruments, Bellevue, WA) was used. After the trocar pierced the trabecular meshwork (TM)/Schlemm’s canal (SC), the delivery button was pressed; however, the stent did not eject but stayed at the tip of the insertion sleeve (Fig. 1a; Video 1). Because the stent moved back into the sleeve after the button was released (Fig. 1b), implantation was attempted again. By pressing the button a second time, the stent was ejected but did not remain at the TM (Fig. 1c, arrow). The dislocated stent on the iris was removed using capsulorhexis forceps (Inami, Tokyo, Japan) (Fig. 1d; Video 2). Observation by surgical microscopy showed that the two stents were connected to each other (Fig. 1e; Video 3). The two stents then were implanted at the desired sites using another injector (Fig. 1f; Video 4). Except for mild hyphema, no postsurgical complication occurred.

**Fig. 2:**
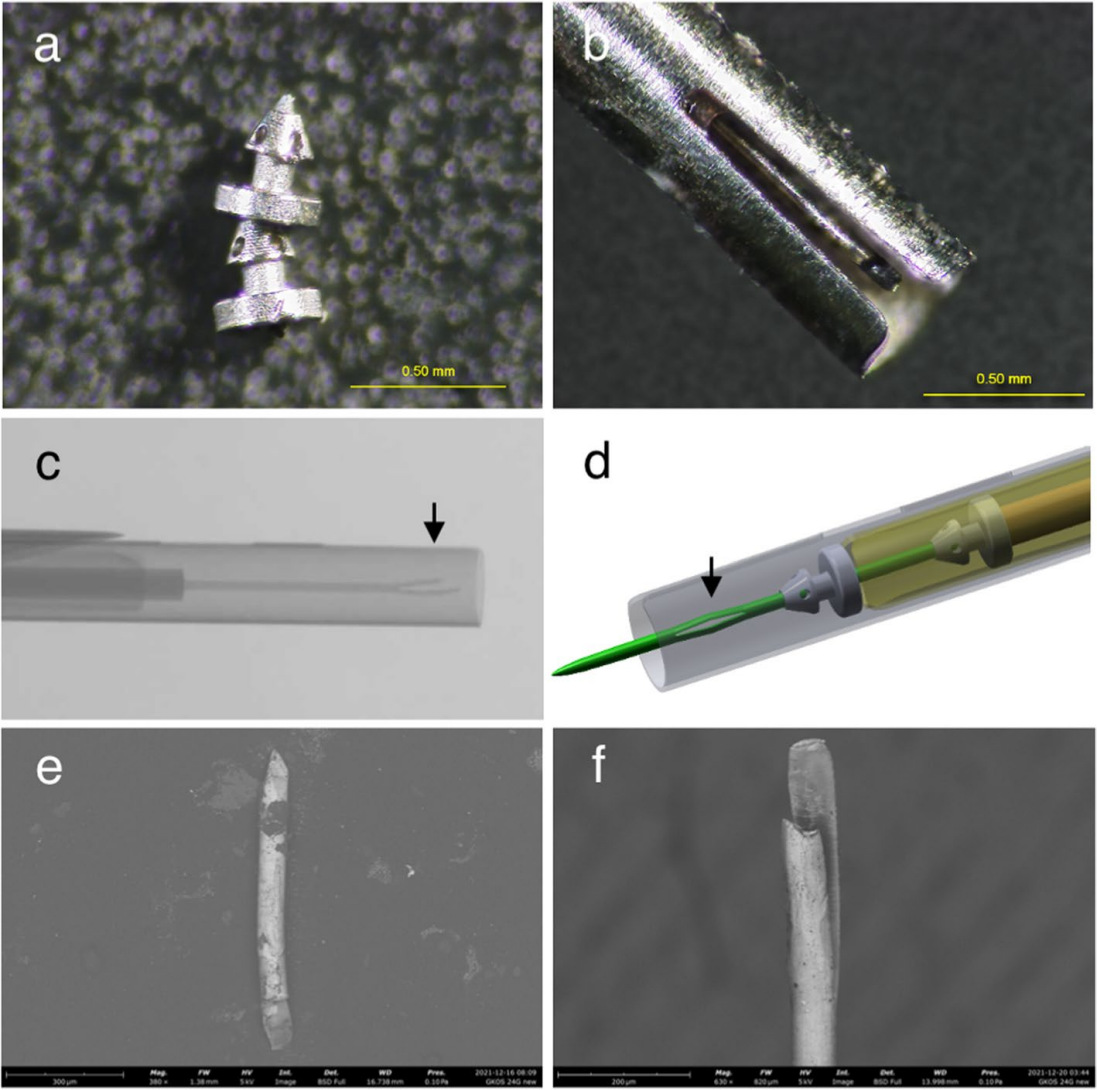
Laboratory inspections of stents and injector. Stereo microscopy images of the explanted stents (**a**) and broken trocar **b**. X-ray image of broken trocar (arrow) **c**. Illustration of trocar structure (arrow) **d**. Scanning electron microscopy images of explanted trocar tip (**e**) and broken trocar **f**. C-F are provided by Glaukos Corporation

Observation of the explanted device under the multi-angle stereomicroscope showed that the two stents seemed to be connected by the broken trocar shaft (Fig. 2a and b). The device was returned to the company (i.e., Glaukos Corporation) for further inspection. An X-ray showed that the trocar shaft was broken at the part referred to as the “sprayed trocar” (Fig. 2c and d, arrows). By scanning electron microscopy observation of the broken trocar shaft (Fig. 2e and f), based on the report provided by the company, the surface features indicated tensile failure. Four months postoperatively, the BCVA was 0.3 OD without correction and 0.8 OS with − 1.25 D astigmatic correction; the IOP was 14 mmHg OU with 2 classes of medications.

## Discussion and conclusion

Dislocation or non-ejection of a stent is possible during iStent inject surgery; therefore, the injector is designed to fire four times. In the current case, because the stent was not ejected during the first deployment, a second deployment was attempted and the two stents connected by the broken tip of the trocar were ejected. This intraoperative complication has not been reported previously.

The manufacturer’s quality engineering team suspected that the trocar was biased during the deployment of the first stent, and during the second deployment, the second stent could have collided with the already bent trocar, thus introducing the trocar to break. A review of the surgical video showed that although most of the trocar appeared to already had been destroyed during the first deployment, this scenario seemed reasonable. The injector was not kept straight during the deployment of the first stent and it might cause non-ejection of iStent and bent trocar shaft. The flange of the *inject* W wider than the *inject* might associate with the trouble in ejecting the stents from the sleeve. In this case, the damage appeared at the “sprayed trocar” where the trocar branched to maintain the stent inside the sleeve. If inspection of the trocar was conducted after the first deployment, the surgeon might notice something unusual. Because the damage occurred at the presumably structurally weak part, this also may happen in other cases. To ensure patient safety, we recommend that surgeons inspect the device when the deployment of either a first or second stent is unsuccessful during an iStent *inject* surgery.

## Supplementary Information

**Additional file 1: Video 1. **First and second deployments of the stents.

**Additional file 2: Video 2. **Removal of dislocated stents.

**Additional file 3: Video 3. **Observation of the explanted stents under a surgical microscope.

**Additional file 4: Video 4. **Implantation of stents using another injector.

## Data Availability

All data generated or analyzed during this study are presented in this article. Further enquiries can be directed to the corresponding author.
